# Predicting hyperopic reserve in children based on school-based vision programs with ensemble learning

**DOI:** 10.3389/fpubh.2026.1910531

**Published:** 2026-08-25

**Authors:** Jing Jiang, Yu Lu, Meng Li, Yi Sun, Rong Lin, Rong Liu, Ran Qin, Qiuxia Chen, Xiao Yang, Yabin Qu

**Affiliations:** 1 Environmental and School Health Department, Guangdong Provincial Center for Disease Control and Prevention, Guangzhou, Guangdong, China; 2 School Health Department, Guangzhou Center for Disease Control and Prevention, Guangzhou, Guangdong, China; 3 School Health Department, Beijing Center for Disease Prevention and Control, Beijing, China; 4 State Key Laboratory of Ophthalmology, Zhongshan Ophthalmic Center, Sun Yat-sen University, Guangzhou, Guangdong, China

**Keywords:** children, hyperopic reserve, machine learning, myopia, predictive model, public health

## Abstract

**Purpose:**

To develop a predictive model based on school-based vision screening programs for evaluating hyperopic reserve status in children aged 5–10 years.

**Methods:**

This cross-sectional study included 8,035 students aged 5–10 years from senior kindergarten and primary school in Guangdong Province, China. Participants underwent ocular biometric measurements, autorefraction, and questionnaire assessment before cycloplegia, after which autorefraction was repeated. An ensemble learning model was constructed to predict low hyperopic reserve (LHR), a refractive status below age-appropriate norms but not yet myopic, using NCR ocular parameters and questionnaire data. Model performance was evaluated and compared through nested 5-fold cross-validation. Sensitivity and robustness analyses were conducted to verify the comprehensive model performance. SHapley Additive exPlanations (SHAP) was utilized to interpret feature contributions and model logic.

**Results:**

On the test set, the ensemble learning model achieved an area under the curve (AUC) of 0.807, an accuracy of 0.737, and a precision of 0.747, outperforming all the single machine learning models. Compared to the direct NCR-based assessment and the +0.50 D / +0.75 D fixed-offset benchmarks (accuracy = 0.614–0.669, precision = 0.593–0.683), the ensemble learning model improved accuracy by 0.068–0.123 and precision by 0.064–0.154. SHAP analysis identified axial length to corneal radius ratio (SHAP value = 0.652), spherical equivalent (SHAP value = 0.514), and school type (SHAP value = 0.312) as the most three important predictors.

**Conclusions and Relevance:**

The proposed ensemble learning model offers a noncycloplegic, convenient approach for school-based vision programs to predict LHR in children aged 5–10 years. This model and its risk-assessment tool may support existing school-based vision programs in preserving children's hyperopic reserve.

## 1 Introduction

Myopia has raised significant international concern in recent decades because of its rapidly increasing prevalence and incidence worldwide (1). This phenomenon is particularly pronounced in China, where a substantial and potentially escalating epidemic of myopia among children and adolescents is underway, projected to affect 63.1% by 2050, including 17.6% with high myopia (2). Since the concept of pre-myopia was introduced in 2019 (3), numerous studies have indicated that early preventive interventions are crucial during the pre-myopic stage (4–6). Notably, recent evidence supports an expanded scope of pre-myopia, with studies increasingly centered on hyperopic reserve, which was identified as a major focus in the 2025 International Myopia Institute Digest (7). Hyperopic reserve refers to the age-appropriate level of hyperopia that provides a protective buffer against myopia development. It has been identified as a strong predictor of future myopia onset (8, 9). Furthermore, accumulating evidence indicates that greater hyperopic reserve in childhood is associated with a lower risk of developing myopia or high myopia later in life (10, 11). However, a nationwide survey in China found that hyperopic reserve among children and adolescents aged 5–18 years was substantially insufficient, with the mean spherical equivalent (SE) reaching−0.50 D by 11 years of age (12).

School-based vision health programs, including visual acuity assessment and noncycloplegic refraction (NCR), have been widely implemented in China since 2021 (13). In these screening programs, children identified as myopic typically receive timely attention and intervention, whereas non-myopic children at high risk of myopia are often overlooked. Accordingly, a promising strategy is to leverage large-scale school screenings to evaluate hyperopic reserve and provide timely intervention for high-risk non-myopic individuals with low hyperopic reserve (LHR). However, a major challenge impedes the implementation of this strategy. LHR, defined as hyperopia below the age-appropriate norm but not yet myopic (13), requires cycloplegic refraction (CR) for accurate measurement. In practice, however, conducting CR in school screenings poses significant challenges, including the scarcity of cycloplegic drops and optometrists, the additional time and effort required, and potential adverse effects (14, 15). Despite these challenges, CR is especially important for pediatric populations because of their active accommodative response (16, 17). It is well recognized that NCR may induce a myopic shift, including overestimation of myopia and underestimation of hyperopia. For instance, Hu et al. found a 0.78 ± 0.79 D difference between cycloplegic and noncycloplegic refraction in 6,364 children (18). A systematic review of 31 studies concluded that NCR tends to produce a myopic shift of 1–2 D relative to cycloplegic measurements in children (19). This methodological discrepancy limits the reliability of hyperopic reserve assessment in school-based programs. Therefore, it is essential to develop a tool that can identify non-myopic children with LHR within school vision programs.

In recent years, machine learning (ML) models have shown considerable potential in ophthalmology and vision research. Previous studies have made progress in predicting the onset of myopia (1, 20), progression of myopia (10, 21), high myopia (22), and pathological myopia (23, 24). Nevertheless, practical predictive models for identifying non-myopic children at high risk of myopia remain limited. Furthermore, most studies employ a single model for prediction, such as linear models, tree-based models, neural network models, etc. If multiple models are integrated for training, theoretically, the overall predictive performance can be improved. Therefore, exploring the performance and applicability of ensemble learning models in practical applications holds considerable significance (25). To bridge this gap, we developed and validated an ensemble learning-based model to predict LHR in children aged 5–10 years without cycloplegia. This interpretable model is expected to achieve better predictive performance than conventional ML models and has the potential to support existing school-based vision programs in preserving children's hyperopic reserve.

## 2 Methods

### 2.1 Study design and participants

**Figure 1:**
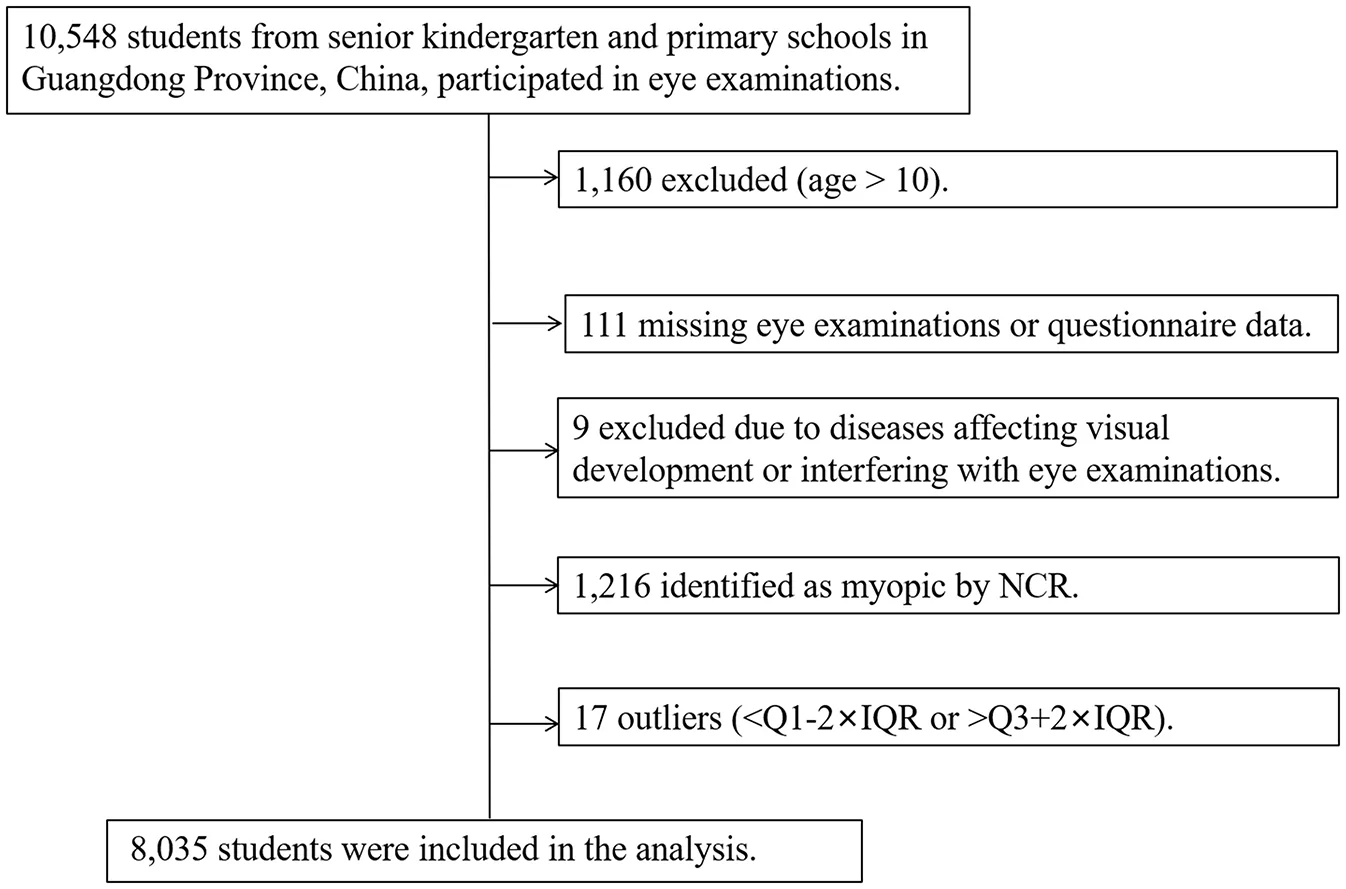
Study participant flowchart. To minimize the influence of extreme outliers on the analytical results, we excluded values that fell below Q1–2 × IQR or exceeded Q3+2 × IQR. Q1 and Q3 represent the first and third quartiles of the age-specific sample data, respectively, and IQR denotes the interquartile range.

**Figure 2:**
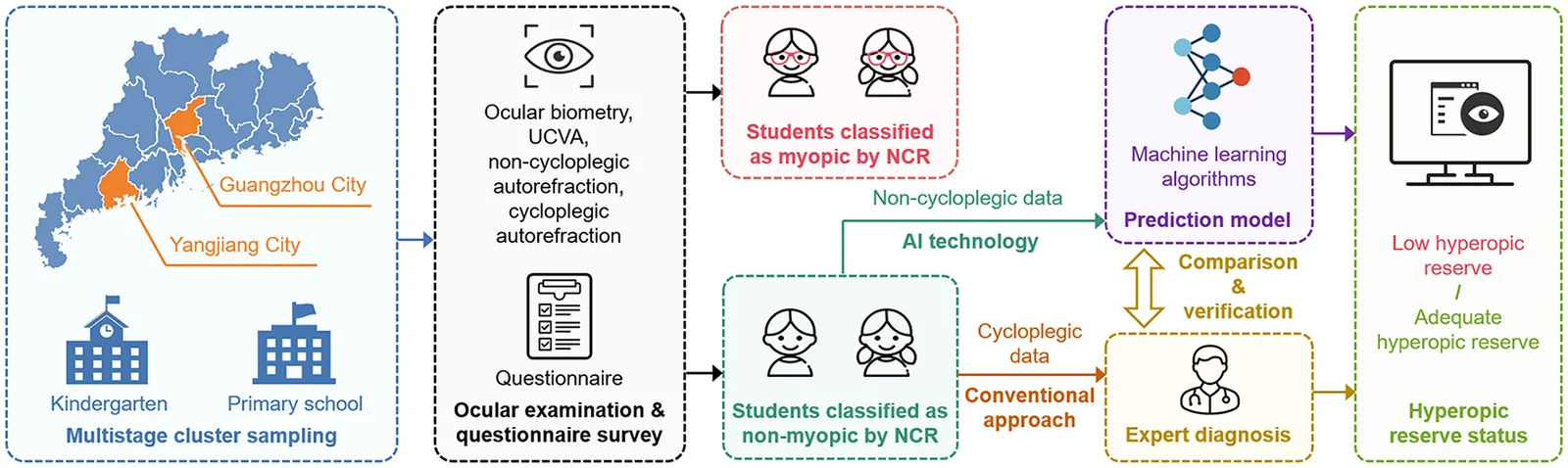
Research framework of the study.

Since 2020, a national hyperopic reserve monitoring project, led by the Beijing Center for Disease Prevention and Control, has been implemented in China. As part of the nationwide initiative, a school-based cross-sectional study on hyperopic reserve in children and adolescents was conducted in Guangdong Province from September 2023 to June 2024. Details of the study have been reported in previous publications (12, 26). Using a multistage cluster sampling method, we recruited 10,548 eligible students from senior kindergarten and primary school (grades 1–6) across two cities (one economically developed and the other less developed) in Guangdong Province. Participants with vision-impairing conditions and those with incomplete examinations or questionnaires were excluded. Finally, 8,035 participants aged 5–10 years identified as non-myopic by NCR were included in the final analysis. The selection process is detailed in Figure 1, and the overall study framework is illustrated in Figure 2. Written informed consent was obtained from the parents or legal guardians of all participants after the study purpose and procedures were explained in detail. This study was performed in accordance with the Declaration of Helsinki and relevant guidelines and regulations. As part of the national hyperopic reserve monitoring project, the study received ethics approval from the institutional review board of Beijing Center for Disease Prevention and Control (2022 No.24).

### 2.2 Questionnaire

A questionnaire was used to collect data on demographic characteristics, visual behavior, and other risk factors associated with myopia in children. The questionnaire was completed jointly by parents and their children in grade 4 and below, and independently by older students. The questionnaire showed good internal consistency (Cronbach's α of 0.81).

### 2.3 Measurements

All participants underwent a comprehensive ocular examination according to standard protocols (27). Prior to cycloplegia, the baseline examination included uncorrected visual acuity (UCVA), ocular biometry and noncycloplegic autorefraction (KR800, Topcon, Tokyo, Japan). Cycloplegia was induced by instilling one drop of 0.5% tropicamide in each eye every 5 min for a total of four doses. Cycloplegic autorefraction was performed 30 min after the final instillation. Full cycloplegia was assumed when the pupil dilated to 6 mm or more and the light reflex was absent. SE was calculated as sphere power (D) + 0.5 × cylinder power (D). Myopia was defined as the concurrent presence of an SE ≤ −0.50 D on NCR and UCVA: ≥ 0.05 LogMAR for children aged 5–6 years, and ≥ 0.00 LogMAR for those older than 6 years (13). According to the recommended standards of the Chinese National Health Commission (28), LHR was defined as a SE below the lower limit of the age-specific reference range. Specifically, the thresholds were set at: +1.50 D for 5 years old, +1.00 D for 6–8 years old, +0.88 D for 9 years old, and +0.75 D for 10 years old. Only right eyes were included for data analysis, because the cycloplegic SE for the right and left eyes were highly correlated (Spearman's correlation coefficient = 0.838).

### 2.4 Machine learning models

**Figure 3:**
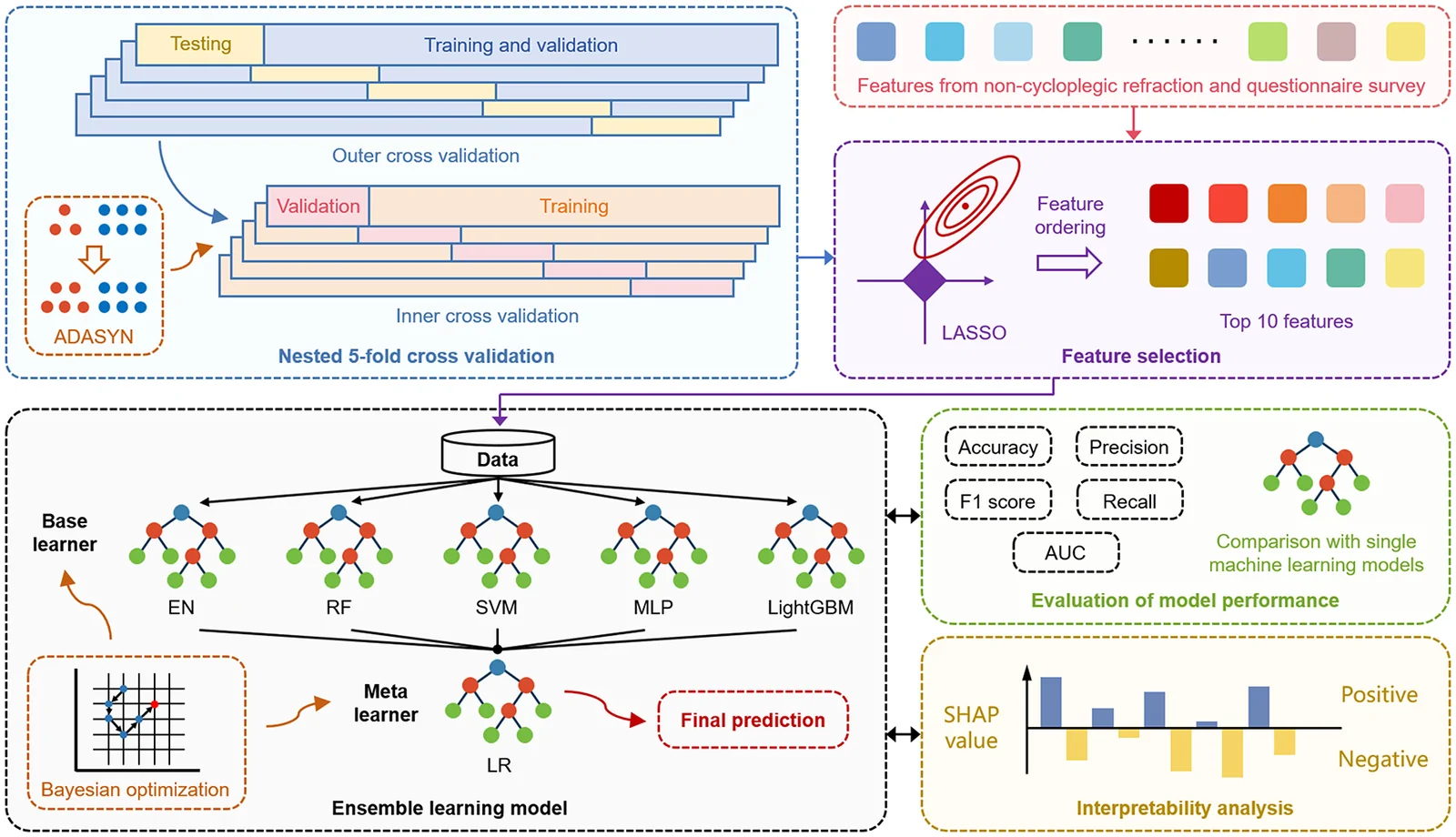
Schematic diagram of the proposed ensemble learning model.

We aimed to develop a machine learning-based model to predict hyperopic reserve status. The binary outcome, namely adequate hyperopic reserve (AHR) or LHR, served as the ground truth for model training and evaluation. Figure 3 illustrates the schematic diagram of the proposed ensemble learning model. Initially, a total of 22 candidate variables derived from ocular examinations and questionnaire data were included as predictors, as shown in Table 1. Remaining isolated missing numerical and categorical predictors were imputed using the median and the most frequent category, respectively. A nested 5-fold cross-validation was implemented, in which the dataset was first divided into 5 outer folds for model evaluation, and within each outer training and validation set, an additional 5-fold inner cross-validation was performed for feature selection and hyperparameter optimization (29, 30). Least absolute shrinkage and selection operator (LASSO) regression was applied within the training set to rank the importance of the features and select the top 10 features for subsequent model development (31). To improve representation of locally underrepresented and difficult-to-classify observations, the adaptive synthetic (ADASYN) oversampling method was applied exclusively to the training data (32).

**Table 1:** Original candidate variables.

Category	Visual behavior	Demographic and parental factors	Noncycloplegic ocular parameters
Variables	Weekly extracurricular tutoring time (Tutoring time)	Age	Uncorrected visual acuity (UCVA)
	Daily time spent outdoors (Outdoor time)	Sex	Spherical power (S)
	Daily after-school homework time (Homework time)	School type	Cylindrical power (C)
	Daily screen time (Screen time)	Body mass index (BMI)	Spherical equivalent (SE)
	Average daily sleep duration (Sleep duration)	Maternal myopia	Axial Length/Corneal Radius ratio (AL/CR)
	Reading or screen use in a lying position (Near work in lying position)	Paternal myopia	
	TV viewing distance (>3 m) (TV viewing distance)	Mode of delivery	
	Computer viewing distance (>66 cm) (Computer viewing distance)	Premature birth (Prematurity)	
	Frequency of breaks during near work (Breaks during near work)		

A stacking ensemble learning framework was employed for prediction. This framework utilized five base learners: Elastic Net (EN), Random Forest (RF), Support Vector Machine (SVM), Multilayer Perceptron (MLP), and Light Gradient Boosting Machine (LightGBM) (33, 34). Logistic Regression (LR) classifier served as the meta learner to integrate the predictions of the base models (31). For both base and meta models, hyperparameters were optimized using Bayesian Optimization (BO) with the maximum of 100 iterations in the inner cross-validation fold (35). Main hyperparameter search spaces of the models were shown in Supplementary Table 1. Calibration assessment and decision-curve analysis were conducted, and model performance on the outer test set was comprehensively evaluated using accuracy, precision, recall, F1-score, area under the curve (AUC), Brier score, calibration slope and calibration intercept with 95% confidence interval (CI). The performance of the ensemble learning model was compared with the performance of each individual model, all trained within the same hyperparameter search ranges. Finally, the SHapley Additive exPlanations (SHAP) algorithm was applied to provide model interpretability and elucidate the contribution of each feature to the predictions (36, 37).

### 2.5 Sensitivity and robustness analyses

To assess the incremental value and robustness of the model, some sets of additional analyses were performed. First, two simple NCR-based benchmarks were also evaluated by adding fixed offsets of 0.50 D and 0.75 D to noncycloplegic SE. Second, interpretable benchmark models were constructed using LR, LightGBM and the complete ensemble framework with AL/CR alone and with AL/CR plus noncycloplegic SE. Third, analyses with and without ADASYN were carried out. Forth, sensitivity analyses excluding noncycloplegic SE, and excluding all noncycloplegic refractive variables including SE, spherical power (S) and cylindrical power (C), were also conducted. Finally, performance of the ensemble learning model was evaluated within different age strata, and boundary-exclusion analysis was conducted for all age when participants whose cycloplegic SE was within ± 0.25 D of the corresponding age-specific LHR threshold were excluded.

### 2.6 Statistical analysis

Descriptive statistics were presented as mean and standard deviation (SD) for continuous variables, and frequency (percentage) for categorical variables. Intergroup comparisons were performed using independent *t*-tests for normally distributed continuous variables, and Pearson's chi-square tests for categorical variables. A two-tailed *P* value < 0.05 was considered statistically significant. R (version 4.1.2) and Python (version 3.9) were used for data analysis.

## 3 Results

### 3.1 Baseline characteristics of the study population

Among the 8,035 non-myopic children aged 5–10 years, the mean age was 6.56 ± 1.31 years. In total, 3,743 (46.6%) were female, and 4,500 (56.0%) were identified as having LHR. For the right eyes, the mean UCVA, AL, noncycloplegic SE and cycloplegic SE were 0.05 ± 0.24 LogMAR, 22.74 ± 0.74 mm, 0.10 ± 0.78 D and 1.01 ± 0.75 D, respectively. The mean differences in SE between cycloplegic and noncycloplegic measurements were 0.64 D in the LHR group, and 1.25 D in the AHR group. Detailed baseline characteristics comparing individuals with AHR and LHR are presented in Table 2.

**Table 2:** Baseline Characteristics of the individuals included in the analysis.

Variable	Overall	Low hyperopic reserve	Adequate hyperopic reserve	*P* value
All subject (number)	8,035 (100%)	4,500 (56.0%)	3,535 (44.0%)	—
Sex (Female, %)	3,743 (46.6%)	2,029 (45.1%)	1,714 (48.5%)	0.002^*^
School type (kindergarten, %)	2,930 (36.5%)	1,677 (37.3%)	1,253 (35.4%)	0.092
Age (year)	6.56 (1.31)	6.62 (1.44)	6.49 (1.12)	< 0.001^*^
BMI (kg/m^2^)	15.53 (2.13)	15.63 (2.21)	15.40 (2.01)	< 0.001^*^
UCVA (LogMAR)	0.05 (0.24)	0.04 (0.26)	0.06 (0.20)	0.001^*^
AL (mm)	22.74 (0.74)	22.92 (0.71)	22.51 (0.73)	< 0.001^*^
Noncycloplegic SE (D)	0.10 (0.78)	−0.07 (0.66)	0.32 (0.86)	< 0.001^*^
Cycloplegic SE (D)	1.01 (0.75)	0.57 (0.46)	1.57 (0.69)	< 0.001^*^

Data are presented as n (%) or mean (SD), ^*^P < 0.05.

### 3.2 Feature selection

**Figure 4:**
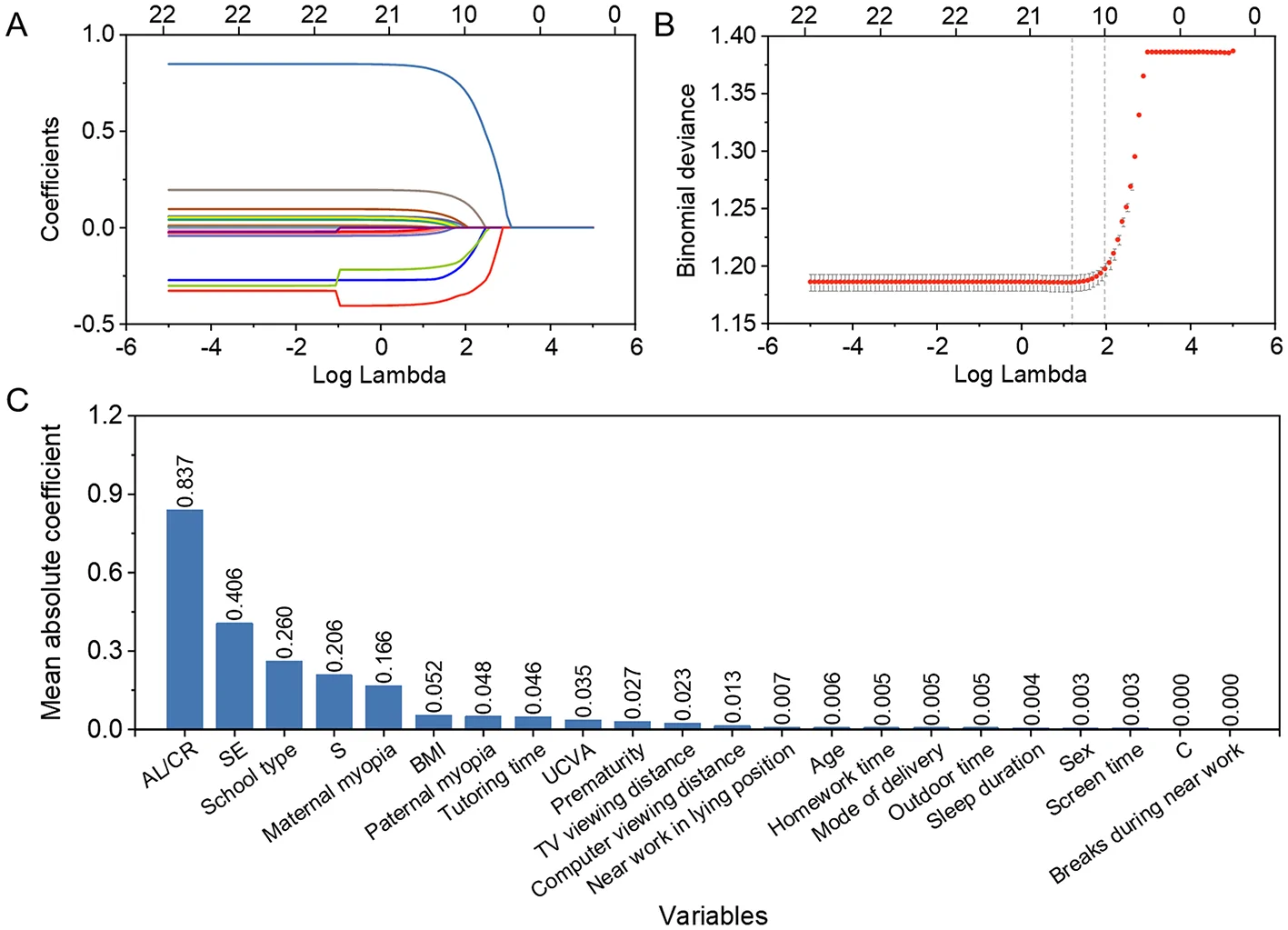
Results of the LASSO regression: **(A)** typical coefficient path plot, **(B)** cross-validation results and **(C)** variable order based on mean absolute coefficient.

The most informative features for LHR prediction were identified through the LASSO regression model, as shown in Figure 4. The regularization path demonstrated stable convergence, and the cross-validated binomial deviance reached its minimum at the optimal penalty parameter. The model retained 10 predictor variables, as the binomial deviance remained within one standard error of the minimum, indicating little loss of model fit despite the substantial reduction in model complexity. The selected features, ranked in descending order of importance, were as follows: AL/CR, SE, school type, S, maternal myopia, BMI, paternal myopia, tutoring time, UCVA, and prematurity.

### 3.3 Comparison of the model performance

**Figure 5:**
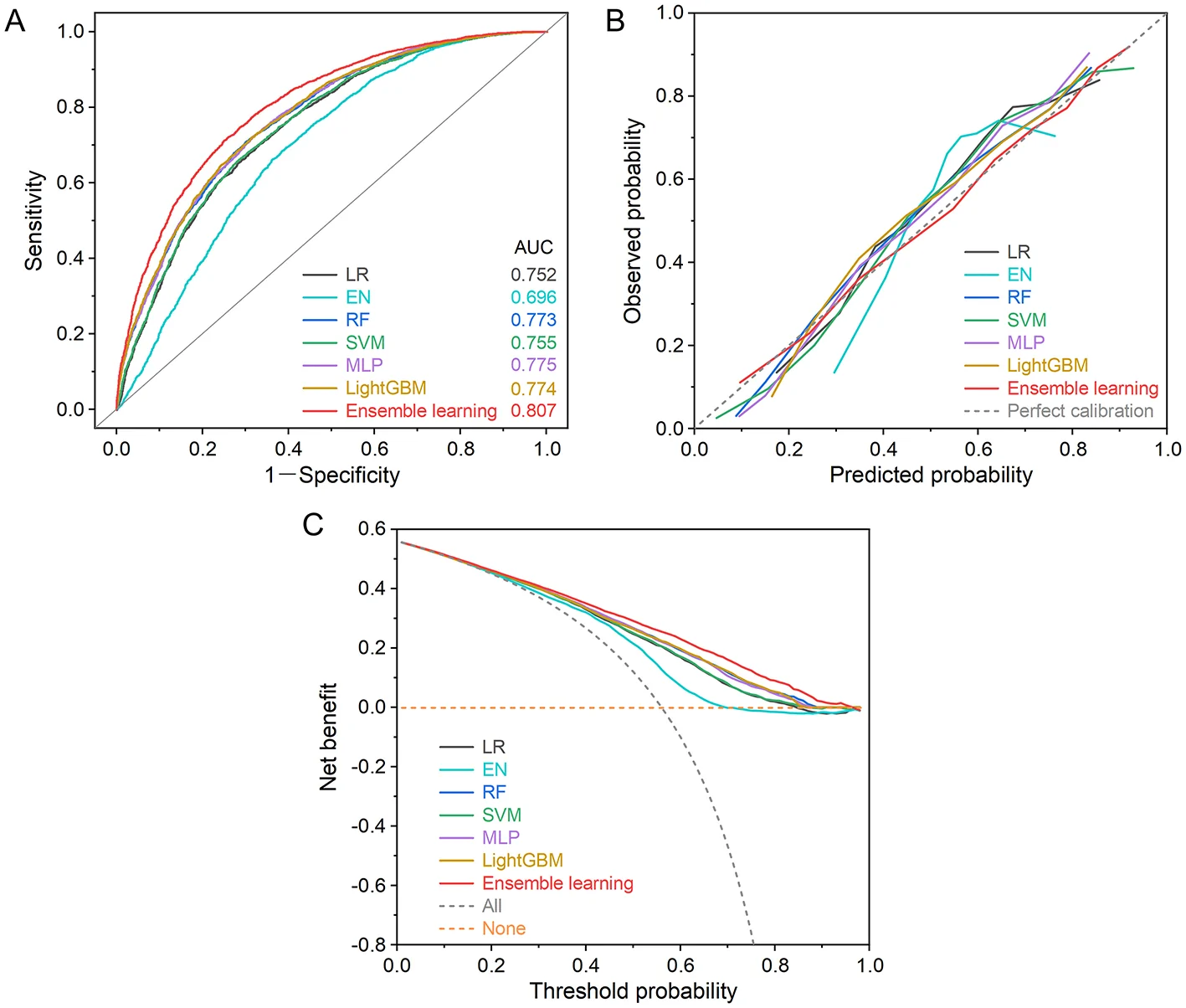
Performances of the models: **(A)** ROC curves, **(B)** calibration plot and **(C)** decision-curves.

The performance comparison of the models on the test set was presented in Table 3 and Figure 5. Among all single ML models, MLP achieved the best performance on the outer test set, achieving an accuracy of 0.708, a precision of 0.733 and an AUC of 0.775. The ensemble learning model outperformed all single ML models, achieving an accuracy of 0.737, a precision of 0.747 and an AUC of 0.807. Compared with MLP, the ensemble learning model improved accuracy by 0.029, precision by 0.014 and AUC by 0.032.

**Table 3:** Performance of the predictive models.

Models	Accuracy (95% CI)	Precision (95% CI)	Recall (95% CI)	F1-score (95% CI)	AUC (95% CI)	Brier (95% CI)	Calibration slope (95% CI)	Calibration intercept (95% CI)
LR	0.687 (0.680, 0.695)	0.724 (0.718, 0.729)	0.715 (0.697, 0.732)	0.719 (0.710, 0.729)	0.752 (0.738, 0.767)	0.201 (0.197, 0.206)	1.123 (0.970, 1.275)	0.159 (0.128, 0.189)
EN	0.655 (0.650, 0.660)	0.688 (0.685, 0.692)	0.701 (0.694, 0.707)	0.695 (0.690, 0.699)	0.696 (0.685, 0.707)	0.223 (0.220, 0.225)	1.307 (1.111, 1.503)	0.140 (0.130, 0.151)
RF	0.705 (0.687, 0.723)	0.742 (0.730, 0.754)	0.726 (0.700, 0.753)	0.734 (0.715, 0.753)	0.773 (0.760, 0.785)	0.192 (0.187, 0.198)	1.092 (0.975, 1.210)	0.135 (0.116, 0.153)
SVM	0.690 (0.677, 0.704)	0.723 (0.713, 0.734)	0.724 (0.697, 0.751)	0.723 (0.708, 0.739)	0.755 (0.736, 0.775)	0.200 (0.193, 0.207)	1.203 (1.063, 1.344)	0.149 (0.111, 0.187)
MLP	0.708 (0.696, 0.719)	0.733 (0.717, 0.749)	0.752 (0.731, 0.772)	0.742 (0.733, 0.752)	0.775 (0.764, 0.786)	0.192 (0.188, 0.196)	1.233 (1.149, 1.318)	0.141 (0.093, 0.189)
LightGBM	0.704 (0.691, 0.718)	0.743 (0.733, 0.754)	0.720 (0.697, 0.743)	0.732 (0.717, 0.746)	0.774 (0.764, 0.784)	0.193 (0.189, 0.197)	1.103 (1.001, 1.205)	0.137 (0.117, 0.156)
Ensemble learning	0.737 (0.727, 0.746)	0.747 (0.742, 0.753)	0.800 (0.787, 0.813)	0.773 (0.764, 0.782)	0.807 (0.795, 0.820)	0.179 (0.174, 0.184)	1.000 (0.873, 1.127)	0.000 (-0.047, 0.046)

If raw noncycloplegic SE values were directly used to determine LHR, the accuracy and precision were only 0.614 and 0.593, respectively. The accuracy and precision of the benchmark with 0.50 D offset to noncycloplegic SE achieved 0.669 and 0.649, whereas those of the benchmark with 0.75 D offset were 0.663 and 0.683, respectively. The ensemble learning model yielded substantial improvements over the two fixed-offset benchmarks, increasing accuracy by 0.068–0.074 and precision by 0.064–0.098.

The ensemble learning model demonstrated favorable internal calibration, with a Brier score of 0.179, a calibration slope of 1.000, and a calibration intercept of −0.0004. The calibration curve approximated the ideal 45° line (Figure 5B). Decision curve analysis demonstrated a positive net benefit across the majority of threshold probabilities, surpassing that of all single models (Figure 5C).

**Figure 6:**
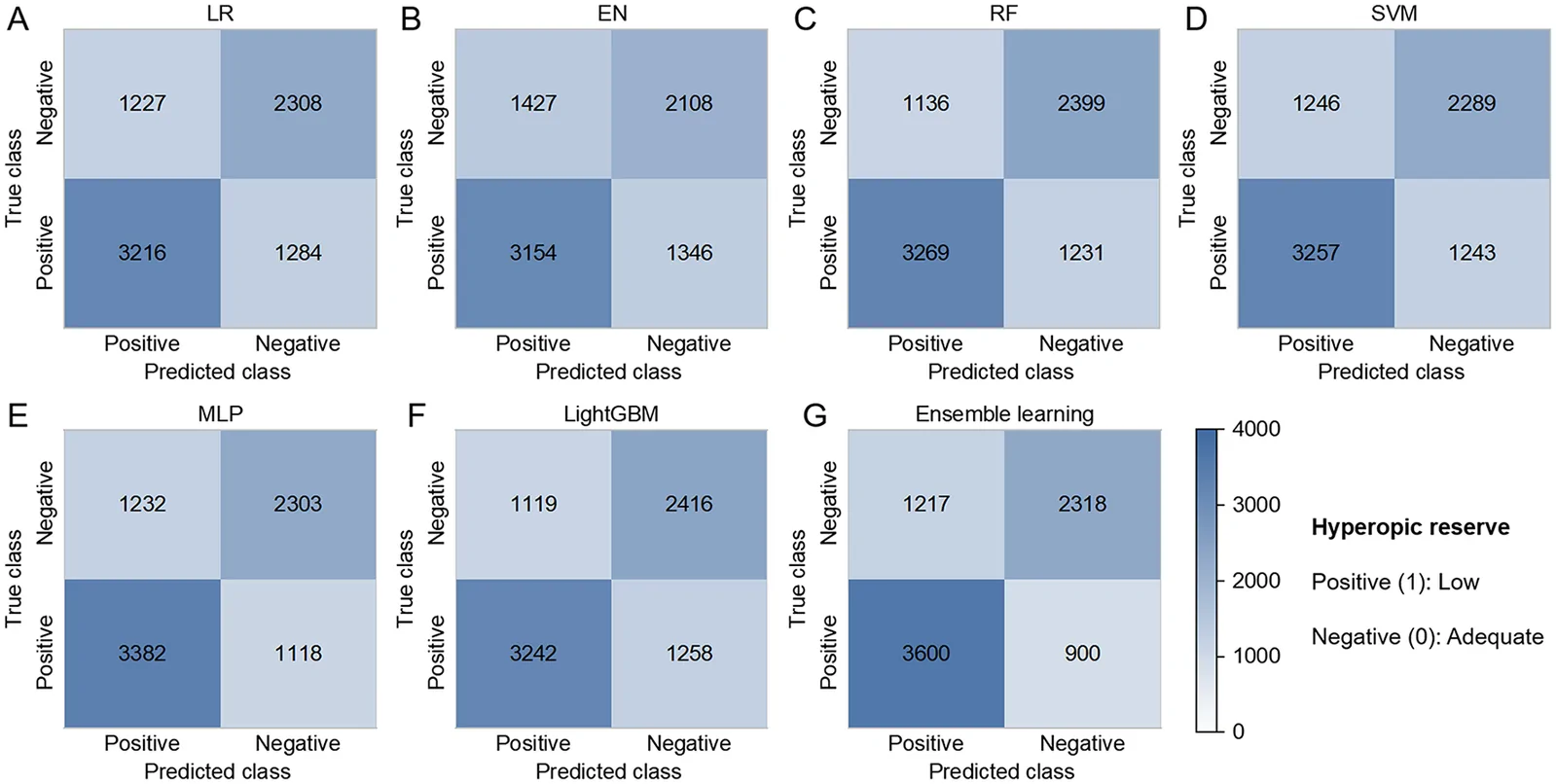
Confusion matrices for the models: **(A)** LR, **(B)** EN, **(C)** RF, **(D)** SVM, **(E)** MLP, **(F)** LightGBM, and **(G)** ensemble learning.

Figure 6 presents the confusion matrices across the 5-fold cross-validation. The ensemble learning model showed the best balance between sensitivity and specificity, with the highest true-positive rate and the lowest false-negative rate across the 5-fold cross-validation process. In contrast, linear models (EN and LR) showed relatively poor sensitivity, while tree-based models (RF and LightGBM) tended toward underdiagnosis.

### 3.4 Sensitivity and robustness of the models

The LR, LightGBM and complete ensemble framework achieved AUCs of 0.697, 0.697, and 0.696 with AL/CR alone, and 0.737, 0.748, and 0.758 with AL/CR plus NCR-SE, respectively, inferior to most single ML models. The full ensemble learning model achieved an improvement of 0.070, 0.059, and 0.049 in AUC, compared to the models with AL/CR plus NCR-SE. Removal of noncycloplegic SE and all noncycloplegic refractive variables reduced AUCs to 0.778 and 0.757 (Supplementary Table 2). ADASYN slightly improved overall classification performance without affecting internal calibration (Supplementary Table 3). Age-stratified AUCs of the ensemble learning model were 0.787, 0.769, 0.770, 0.803, 0.821, and 0.796 among children aged 5, 6, 7, 8, 9 and 10 years, respectively (Supplementary Table 4). In the boundary-exclusion analysis, the ensemble learning model achieved an AUC of 0.856 and an accuracy of 0.778.

### 3.5 Interpretability analysis

**Figure 7:**
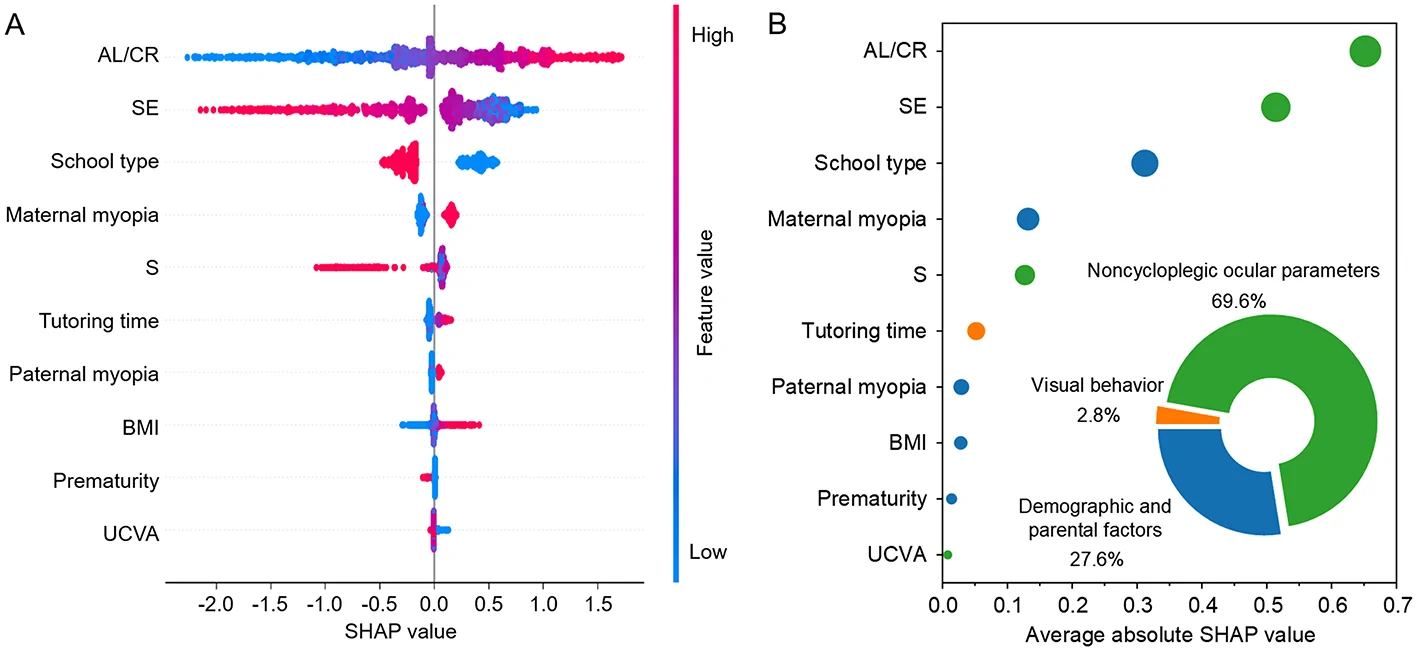
Results of interpretability analysis: **(A)** SHAP summary plot and **(B)** feature contributions.

The contributions and effects of the variables were quantified and compared through interpretability analysis, as shown in Figure 7A. SHAP analysis revealed AL/CR (0.652), SE (0.514) and school type (0.312) as the three most important variables. When features were aggregated into broader categories, noncycloplegic ocular parameters primarily drove the predictive performance, accounting for 69.6% of the total contribution, as shown in Figure 7B.

### 3.6 Development of the predictive software tool

**Figure 8:**
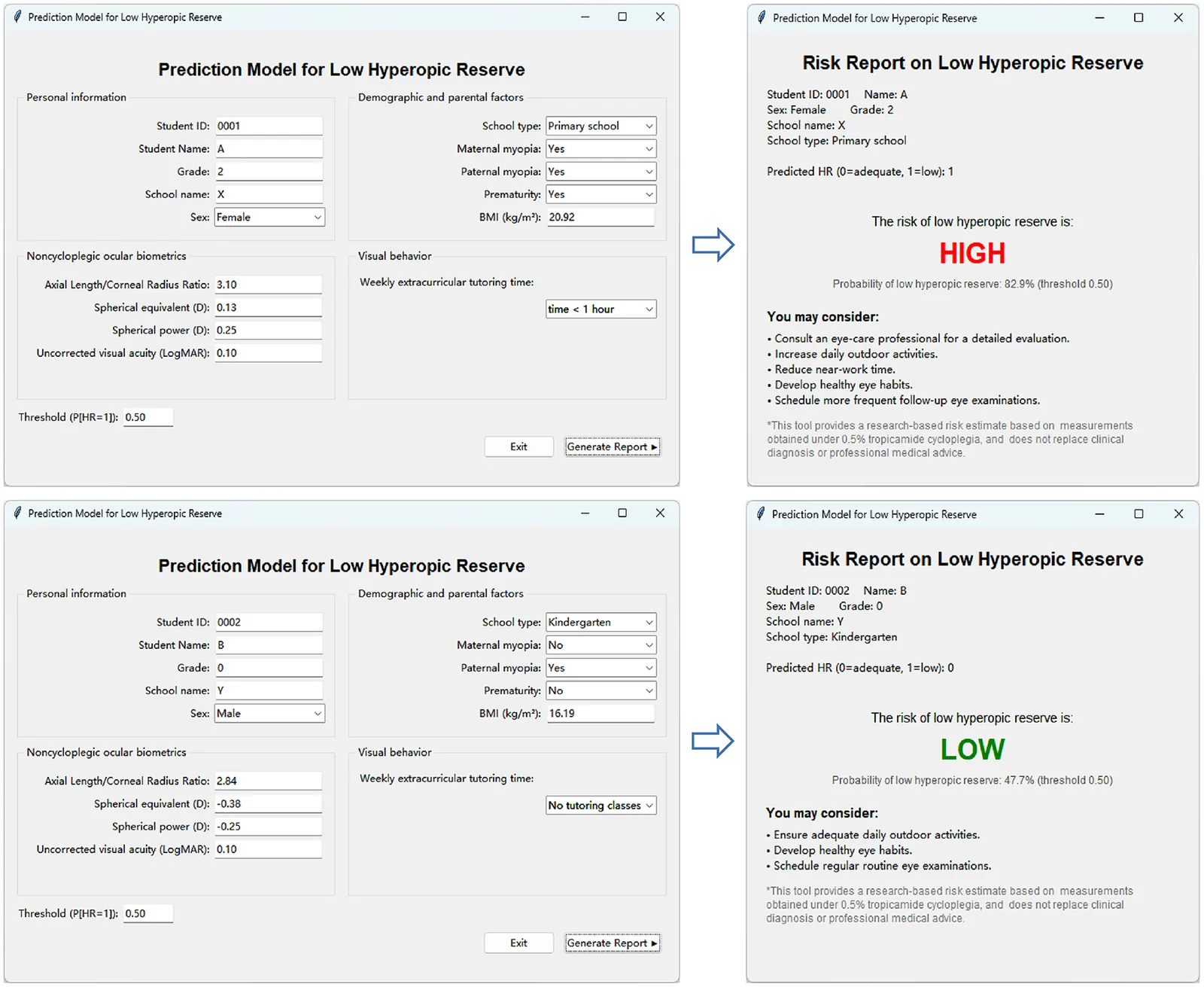
The interface of low hyperopic reserve prediction software.

A predictive software tool was developed based on the optimized ensemble learning model. The user interface allowed practitioners to input the 10 key predictive variables, as shown in Figure 8. Based on these inputs, the system could calculate and output the individual's probability of having LHR. Furthermore, the software could automatically classify the subject as either “high” or “low” risk and provide targeted screening recommendations accordingly. For illustrative visualization, a probability threshold of 0.50 was predefined to display risk classifications, and users may adjust the threshold according to the intended screening context.

## 4 Discussion

In this study, we developed and validated an ensemble learning model capable of predicting LHR in non-myopic children. This model may have significant practical value for school-based vision programs and public health settings for several reasons. First, it targets children aged 5–10 years who are in a critical window for myopia prevention. In this age group, active accommodation often causes substantial discrepancies between noncycloplegic screening results and true refractive status. Second, the model is designed for real-world applications. It uses noncycloplegic data that are readily obtainable in large-scale student vision screenings. Third, it helps remind parents that their children may have “high” risk of LHR for their age, enabling reinforced preventive measures, including consulting an eye-care professional for a detailed evaluation, increasing outdoor activities, reducing near-work time, developing healthy eye habits, and more frequent follow-up monitoring. This practical tool may support school-based vision programs in preserving children's hyperopic reserve.

In recent years, the concept of hyperopic reserve has received increasing international attention (7, 38). Several longitudinal studies have shown that lower levels of baseline hyperopia are associated with a higher risk of subsequent myopia. In a follow-up of the Sydney Myopia Study, myopia risk increased with decreasing baseline refraction. In the younger cohort (mean age 6 years), children with baseline refraction ≤ +1.00 D had a higher incidence of myopia than those with ≥ +2.00 D (39). Similarly, a Chinese study including 2,628 non-myopic first-grade students reported that the 5-year cumulative incidence of myopia increased progressively as baseline hyperopic reserve decreased, reaching 4.6%, 26.3%, 52.3%, 78.6%, 92.6%, and 94.3% in children with baseline hyperopic reserve of >+2.00 D, +1.50 to +2.00 D, +1.00 to +1.50 D, +0.50 to +1.00 D, 0.00 to +0.50 D, and−0.50 to 0.00 D, respectively (40). This focus findings support increasing attention to earlier, more personalized, and more prevention-oriented approaches to myopia control. The Chinese government has initiated a strategic shift from “reactive treatment” to “proactive prevention,” positioning hyperopic reserve monitoring at the core of its national public health agenda (41). By evaluating hyperopic reserve status, it may be possible to complement traditional post-onset refraction examination to enable earlier and more precise risk stratification and intervention. The strategy of protecting hyperopic reserve in children and adolescents has gained support from government bodies, fostering multi-sectoral collaboration between educational and health authorities (42, 43). A joint circular recommends at least annual vision and refraction exams for kindergarten and primary students, including hyperopic reserve assessment and individual refractive records. For those who are screened and identified with LHR, parents should promptly seek for professional examinations, including cycloplegic refraction (43).

To translate hyperopic reserve strategies into population-wide applications for children, we developed an ensemble learning model tailored for school-based vision screening. The model demonstrated robust predictive performance (AUC of 0.807, precision of 0.747, accuracy of 0.737). Compared to individual ML models, the ensemble learning framework improved the comprehensive performance, while maintaining lower cross-validation variance. This superiority stemmed from the ensemble's ability to capture the highly non-linear and complex relationships between ocular biometrics and true refractive status, which simple linear models were less capable of detecting. Furthermore, while standalone tree-based algorithms were prone to overfitting in health screening datasets, our approach mitigated this by aggregating diverse base learners, optimizing hyperparameters via BO. Compared to the direct NCR-based assessment, and the +0.50 D / +0.75 D fixed-offset benchmarks, the ensemble learning model achieved improvements in both accuracy and precision. From a public health perspective, the model's enhanced sensitivity and its ability to minimize the false-negative rate are particularly crucial for population-level screening.

The additional analyses clarified both the source and magnitude of the model's predictive value. The LR, LightGBM and ensemble learning models with AL/CR alone and with AL/CR plus noncycloplegic SE formed a interpretable predictive foundation, but did not reproduce the performance of the full multivariable ensemble. The complete ensemble learning model showed improved discrimination, classification performance, and Brier score. This improvement stemed from the incremental value of the flexible ensemble modeling framework, as well as incremental information from the remaining ocular, visual behavioral, demographic and parental predictors. The model retained moderate discrimination after all NCR variables were removed, suggesting that its performance was not solely driven by the dependence between noncycloplegic and cycloplegic refractive measurements. Moderate discrimination was retained within each age stratum, and the model's predictive value was confirmed within each exact-age stratum. This demonstrated that the full ensemble captures within-age refractive and biometric heterogeneity rather than relying solely on the age-dependent definition of LHR. Model discrimination and precision improved after observations within ± 0.25 D of the age-specific thresholds were removed, whereas recall remained nearly unchanged. The model retained strong discrimination and favorable calibration among children with measurements more clearly separated from the thresholds. Collectively, these findings demonstrate measurable robustness and incremental predictive value.

Previous research on ML for myopia prediction has reported a wide range of performance, with AUCs varying from approximately 0.67–0.953 (20, 44–48). These discrepancies likely reflect differences in examination methods, target populations, prediction outcomes, study design, and other factors. Studies using clinical data, such as cycloplegic refraction or fundus imaging (20, 44), have shown promising results. For example, a study used cycloplegic refraction data to develop an XGBoost-based model for predicting myopia onset and achieved an AUC of 0.953 (44). Although such performance is not unexpected given the diagnostic precision of these clinical modalities, their dependence on specialized examinations limits their feasibility for large-scale, school-based public health screening. Some studies have explored noncycloplegic data and reported favorable results, with R-squared values of up to 0.927 for predicting cycloplegic SE using an AdaBoost regression model. However, a substantial proportion of participants (60.3%) in that study were already myopic (45). By contrast, school-based research using noncycloplegic methods to identify pre-myopia in non-myopic children remains limited. Only a few studies have examined pre-myopia prediction in younger populations with non-myopic or low-myopia prevalence, reporting AUCs between 0.67 and 0.78 (46–48). In our study, the model was applied to a young, non-myopic population with a mean age of 6.56 years and relatively high hyperopic reserve, and achieved an AUC of 0.807. This result slightly outperformed those reported in previous similar studies and added new evidence to this relatively underexplored area.

SHAP analysis revealed AL/CR as the strongest predictor of LHR. AL/CR has been demonstrated to be significantly more effective for detecting myopia in children than AL or UCVA and could be a useful marker for the onset and progression of myopia in most screening settings (47, 49). As a matter of fact, AL/CR has become a routine measurement in school-based vision programs in countries such as China. Notably, its contribution (0.652) was higher than that of the second-ranked SE (0.514). This discrepancy arises because SE was derived from NCR, where active ciliary muscle accommodation often underestimates hyperopia (19), whereas AL and CR remain independent of accommodation. However, this does not reflect an intrinsic superiority of biometry over refraction. Rather, the predictive advantage of AL/CR is attributable to the myopic shift, which reduces the discriminative power of NCR SE. School type also contributed substantially, which may reflect older age and increased near-work demands. Maternal myopia (0.132) had a stronger impact than paternal myopia (0.029), aligning with previous findings that maternal myopia has a high correlation with children's refractive development (50, 51). Finally, UCVA contributed minimally (0.008). There is evidence that it cannot be used to reliably screen for hyperopia (52). While a child's UCVA appears adequate, additional measurements of ocular morphological and refractive parameters should be performed to assess visual status.

Several limitations should be acknowledged. First, our model is specifically designed for school-based vision programs with access to refraction and ocular biometry. Second, the study included only Chinese children and followed national guidelines, which may limit the generalizability of our findings to some extent. Third, considering safety, rapid recovery, feasibility, and participant acceptability, we used 0.5% tropicamide for cycloplegia. Although we strictly adhered to a standardized protocol, this concentration may result in incomplete cycloplegia and may overclassify LHR. Consequently, our model may not be applicable to settings using other stronger cycloplegic agents. External validation using 0.5% tropicamide in children from different regions and populations is therefore warranted, and we are actively collaborating with other centers to collect external datasets for further model validation. Finally, dichotomization of a continuous cycloplegic SE measurement necessarily may discard information and create label instability near the age-specific thresholds. The establishment of predictive models for continuous cycloplegic SE values have been planned.

## 5 Conclusions

In this study, we developed an ensemble learning model to predict LHR in children aged 5–10 years using noncycloplegic data, providing a practical risk-stratification tool for existing school-based vision programs. The ensemble learning model outperformed all the single ML algorithms, the direct NCR-based assessment, and the fixed-offset benchmarks. Sensitivity and robustness analyses verified its steady performance under different conditions. While this tool cannot replace clinical diagnosis, it may support school-based vision programs in preserving children's hyperopic reserve.

## Data Availability

The raw data supporting the conclusions of this article will be made available by the authors, without undue reservation.
