# Supplementary material for: Predicting hyperopic reserve in children based on school-based vision programs with ensemble learning
Source: Front Public Health. 2026 Aug 25;14:1910531. doi: 10.3389/fpubh.2026.1910531 (PMC13550169; doi:10.3389/fpubh.2026.1910531)
Supplement: Supplementary file 1 [file Table_1.docx]

Supplementary Material

# 1 Supplementary Tables

**Supplementary TABLE 1** Main hyperparameters and search spaces of the models.

| **Models** | **Main hyperparameters and search spaces** |
| --- | --- |
| LR | C: 10^−5^–10^5^  Class Weight: “balanced”, None |
| EN | C: 10^−5^–10^5^  L1 mixing ratio: 0–1 |
| RF | Number of estimators: 100−1,000  Maximum tree depth: 2–56  Minimum samples for node splitting: 2–200  Minimum samples per leaf: 1–200  Number of features considered at each split: “sqrt”, “log2”, None |
| SVM | C: 10^−5^–10^5^  Gamma: 10^−7^–10^5^ |
| MLP | Number of hidden layers: 1–3  Number of neurons in the hidden layer: 32–256  L2 regularization parameter: 10^−6^–10^−1^  Initial learning rate: 10^−6^–10^−1^  Activation function: “relu”, “tanh”, “logistic”  Solver: “adam”, “lbfgs” |
| LightGBM | Number of estimators: 100–1,000  Number of leaves: 16–256  Maximum tree depth: 2–128  Learning rate: 10^−5^–10^−1^  Minimum child samples: 1–200  Row subsampling fraction: 0.5–1.0  Feature subsampling fraction: 0.5–1.0 |
| Ensemble learning | Consistent with single models mentioned above |

**Supplementary TABLE 2** Performance of the models in the robustness analysis.

| **Models** | **Accuracy**  **(95% CI)** | **Precision**  **(95% CI)** | **Recall**  **(95% CI)** | **F1-score**  **(95% CI)** | **AUC**  **(95% CI)** | **Brier score (95% CI)** | **Calibration slope (95% CI)** | **Calibration intercept (95% CI)** |
| --- | --- | --- | --- | --- | --- | --- | --- | --- |
| LR (only AL/CR) | 0.650 (0.640, 0.660) | 0.679 (0.674, 0.685) | 0.709 (0.686, 0.733) | 0.694 (0.681, 0.707) | 0.697 (0.683, 0.712) | 0.218 (0.213, 0.223) | 1.030 (0.895, 1.165) | 0.103 (0.066, 0.140) |
| LR (AL/CR plus noncycloplegic SE) | 0.676 (0.660, 0.692) | 0.715 (0.700, 0.731) | 0.700 (0.684, 0.715) | 0.707 (0.693, 0.722) | 0.737 (0.720, 0.753) | 0.207 (0.202, 0.212) | 1.146 (1.013, 1.279) | 0.175 (0.136, 0.213) |
| LightGBM (only AL/CR) | 0.652 (0.643, 0.662) | 0.677 (0.670, 0.684) | 0.725 (0.702, 0.748) | 0.700 (0.689, 0.712) | 0.697 (0.685, 0.709) | 0.219 (0.215, 0.223) | 1.047 (0.928, 1.166) | 0.115 (0.081, 0.148) |
| LightGBM (AL/CR plus noncycloplegic SE) | 0.685 (0.678, 0.691) | 0.717 (0.708, 0.725) | 0.724 (0.713, 0.735) | 0.720 (0.715, 0.726) | 0.748 (0.741, 0.756) | 0.202 (0.199, 0.205) | 1.139 (1.044, 1.235) | 0.155 (0.119, 0.192) |
| Ensemble learning (only AL/CR) | 0.651 (0.642, 0.661) | 0.654 (0.637, 0.670) | 0.806 (0.767, 0.844) | 0.721 (0.713, 0.729) | 0.696 (0.682, 0.709) | 0.217 (0.212, 0.223) | 0.981 (0.832, 1.130) | 0.002 (-0.057, 0.061) |
| Ensemble learning (AL/CR plus noncycloplegic SE) | 0.694 (0.679, 0.709) | 0.697 (0.686, 0.708) | 0.802 (0.783, 0.820) | 0.746 (0.732, 0.759) | 0.758 (0.747, 0.770) | 0.197 (0.192, 0.202) | 0.997 (0.883, 1.112) | 0.002 (-0.043, 0.046) |
| Ensemble learning (excluding noncycloplegic SE) | 0.710 (0.704, 0.717) | 0.715 (0.706, 0.725) | 0.802 (0.793, 0.811) | 0.756 (0.753, 0.760) | 0.778 (0.768, 0.788) | 0.188 (0.184, 0.193) | 0.997 (0.897, 1.098) | 0.003 (-0.024, 0.029) |
| Ensemble learning (excluding noncycloplegic SE, S and C) | 0.694 (0.685, 0.704) | 0.698 (0.687, 0.710) | 0.800 (0.784, 0.817) | 0.746 (0.739, 0.753) | 0.757 (0.746, 0.768) | 0.198 (0.193, 0.202) | 0.982 (0.877, 1.086) | 0.001 (-0.064, 0.067) |
| Ensemble learning (excluding AL/CR) | 0.692 (0.687, 0.697) | 0.696 (0.683, 0.709) | 0.801 (0.775, 0.828) | 0.744 (0.739, 0.750) | 0.745 (0.740, 0.749) | 0.200 (0.197, 0.203) | 0.968 (0.871, 1.065) | 0.010 (-0.048, 0.068) |

**Supplementary TABLE 3** Performance of the models without ADASYN in the sensitivity analysis.

| **Models** | **Accuracy**  **(95% CI)** | **Precision**  **(95% CI)** | **Recall**  **(95% CI)** | **F1-score**  **(95% CI)** | **AUC**  **(95% CI)** | **Brier score (95% CI)** | **Calibration slope (95% CI)** | **Calibration intercept (95% CI)** |
| --- | --- | --- | --- | --- | --- | --- | --- | --- |
| LR | 0.692 (0.681, 0.704) | 0.704 (0.694, 0.713) | 0.778 (0.763, 0.794) | 0.739 (0.729, 0.750) | 0.753 (0.739, 0.767) | 0.199 (0.195, 0.204) | 1.190 (1.057, 1.323) | -0.002 (-0.037, 0.032) |
| EN | 0.660 (0.653, 0.667) | 0.663 (0.657, 0.669) | 0.799 (0.794, 0.804) | 0.724 (0.719, 0.730) | 0.692 (0.682, 0.702) | 0.220 (0.217, 0.222) | 1.086 (0.953, 1.220) | -0.022 (-0.041, -0.002) |
| RF | 0.708 (0.691, 0.725) | 0.728 (0.717, 0.739) | 0.764 (0.737, 0.791) | 0.745 (0.728, 0.763) | 0.773 (0.759, 0.786) | 0.191 (0.185, 0.197) | 1.016 (0.923, 1.109) | 0.022 (-0.009, 0.053) |
| SVM | 0.693 (0.682, 0.703) | 0.703 (0.694, 0.712) | 0.782 (0.770, 0.794) | 0.740 (0.731, 0.749) | 0.753 (0.739, 0.768) | 0.199 (0.195, 0.204) | 1.194 (1.057, 1.332) | -0.008 (-0.047, 0.031) |
| MLP | 0.707 (0.694, 0.720) | 0.713 (0.702, 0.724) | 0.796 (0.781, 0.812) | 0.752 (0.741, 0.764) | 0.773 (0.757, 0.788) | 0.191 (0.185, 0.197) | 1.138 (1.092, 1.184) | 0.010 (-0.024, 0.044) |
| LightGBM | 0.707 (0.694, 0.721) | 0.726 (0.718, 0.734) | 0.768 (0.746, 0.789) | 0.746 (0.732, 0.760) | 0.776 (0.766, 0.787) | 0.190 (0.185, 0.195) | 1.025 (0.946, 1.104) | 0.020 (-0.011, 0.051) |
| Ensemble learning | 0.731 (0.723, 0.740) | 0.741 (0.731, 0.750) | 0.801 (0.787, 0.814) | 0.769 (0.762, 0.776) | 0.803 (0.792, 0.813) | 0.179 (0.174, 0.184) | 0.996 (0.870, 1.123) | 0.000 (-0.044, 0.044) |

**Supplementary TABLE 4** Performance of the Ensemble learning models within different age strata and in the boundary-exclusion analysis.

| **Age strata** | **Accuracy**  **(95% CI)** | **Precision**  **(95% CI)** | **Recall**  **(95% CI)** | **F1-score**  **(95% CI)** | **AUC**  **(95% CI)** | **Brier score (95% CI)** | **Calibration slope (95% CI)** | **Calibration intercept (95% CI)** |
| --- | --- | --- | --- | --- | --- | --- | --- | --- |
| 5 | 0.747 (0.733, 0.761) | 0.825 (0.805, 0.846) | 0.809 (0.796, 0.822) | 0.817 (0.808, 0.825) | 0.787 (0.766, 0.807) | 0.164 (0.153, 0.174) | 0.964 (0.774, 1.154) | 0.020 (-0.127, 0.168) |
| 6 | 0.667 (0.654, 0.680) | 0.568 (0.551, 0.584) | 0.802 (0.749, 0.854) | 0.664 (0.651, 0.676) | 0.769 (0.746, 0.793) | 0.191 (0.182, 0.201) | 0.956 (0.774, 1.138) | -0.011 (-0.125, 0.103) |
| 7 | 0.692 (0.675, 0.710) | 0.684 (0.668, 0.700) | 0.801 (0.775, 0.827) | 0.738 (0.722, 0.753) | 0.770 (0.743, 0.797) | 0.196 (0.183, 0.208) | 0.985 (0.762, 1.208) | 0.012 (-0.043, 0.067) |
| 8 | 0.745 (0.704, 0.786) | 0.789 (0.750, 0.829) | 0.801 (0.763, 0.840) | 0.795 (0.763, 0.828) | 0.803 (0.757, 0.850) | 0.174 (0.154, 0.193) | 0.965 (0.629, 1.301) | 0.035 (-0.016, 0.086) |
| 9 | 0.773 (0.726, 0.819) | 0.881 (0.853, 0.908) | 0.801 (0.751, 0.852) | 0.839 (0.803, 0.875) | 0.821 (0.760, 0.883) | 0.143 (0.122, 0.163) | 1.088 (0.405, 1.771) | -0.114 (-0.645, 0.417) |
| 10 | 0.789 (0.748, 0.828) | 0.858 (0.813, 0.902) | 0.842 (0.784, 0.900) | 0.849 (0.819, 0.878) | 0.796 (0.743, 0.849) | 0.145 (0.122, 0.167) | 0.773 (0.505, 1.042) | 0.271 (-0.204, 0.745) |
| Boundary-exclusion analysis | 0.778 (0.769, 0.787) | 0.814 (0.795, 0.833) | 0.801 (0.770, 0.832) | 0.807 (0.797, 0.817) | 0.856 (0.846, 0.866) | 0.150 (0.143, 0.157) | 0.978 (0.895, 1.062) | 0.006 (-0.095, 0.108) |
